# Postgraduate training in general practice in Germany: A narrative review

**DOI:** 10.3205/000344

**Published:** 2025-08-25

**Authors:** Caroline Tornow, Julia Freyer Martins Pereira, Elizabeth Mathias, Jean-François Chenot

**Affiliations:** 1 Greifswald University Medical Center, Institute for Community Medicine, Department of General Practice, Greifswald, Germany

**Keywords:** postgraduate training, general practice, German medical system, vocational training, primary care

## Abstract

Postgraduate training varies significantly both between countries and among medical specialties within a nation, due to differences in medical education and health care systems. We aim to provide detailed information about postgraduate training in general practice. This narrative review is based on a selective literature review, supplemented by legal texts and information from official institutions.

Postgraduate training is strongly influenced by Germany’s federal governmental structure, funding regulations and lack of structured programmes attached to academic bodies. This system allows for freedom in career choice, and the organisation of education and training after medical school. However, it presents significant challenges for workforce planning. Additionally, it places a heavy organisational burden on the individual trainee, who is responsible for organising their own rotations and ensuring that all necessary skills are acquired during training. In response to an increasing shortage in general practitioners, recent years have seen new developments such as the establishment of competence centres and coordination offices. Nevertheless, further improvements are necessary to guarantee up-to-date training, so that future general practitioners can meet the growing challenges of medical care.

## Abstract (de)

Die Facharztweiterbildung unterscheidet sich sowohl international als auch national zwischen den medizinischen Fachgebieten erheblich, was auf Unterschiede der medizinischen Ausbildungs- und Gesundheitssysteme zurückzuführen ist. Unser Ziel ist es, die Weiterbildung zur Fachärztin bzw. zum Facharzt für Allgemeinmedizin in Deutschland darzustellen. Die Grundlage dieses Artikels bildet eine systematische Literaturrecherche, die um relevante Gesetzestexte und Informationen öffentlicher Einrichtungen ergänzt wurde.

Die Facharztweiterbildung wird maßgeblich durch die Bundesländer, spezifische Förderbestimmungen sowie den Mangel an strukturierten Weiterbildungsprogrammen beeinflusst. Es wird eine große Freiheit in der Wahl der Facharztrichtung und der Gestaltung der Weiterbildung gewährt, die aber eine ärztliche Bedarfsplanung erschwert. Das führt auch dazu, dass die Verantwortung für die Organisation der notwendigen Rotationen und den Erwerb relevanter Kompetenzen überwiegend in der Eigenverantwortung der Ärztinnen und Ärzten in Weiterbildung liegt.

In Anbetracht des zunehmenden Hausärztemangels wurden in den letzten Jahren einige Maßnahmen ergriffen, wie die Etablierung von Kompetenzzentren und Koordinierungsstellen für die Weiterbildung. Dennoch sind weitere Reformen notwendig, um die zukünftigen Allgemeinmedizinerinnen und Allgemeinmediziner optimal auf die Herausforderungen der kommenden Jahre vorzubereiten.

## Introduction

Postgraduate training of junior doctors does not only differ greatly between countries but also across different specialties. Specifically, in primary care, the role of general practitioners (GPs) in a given health care system affects the required education and training. While past studies have compared training programmes across various countries [1], [2], [3] or provided overviews [4], there has been no recent detailed description of specialty training in general practice in Germany. 

In 2022, there were 42,859 registered GPs in Germany (approx. 10% of all registered physicians in Germany). Additionally, 1,561 outpatient internal specialists and 185 “practical doctors” (praktischer Arzt [5]) were providing primary care [6]. However, over 7,000 GPs (approx. 6%) are over 60 years old and expected to retire within the next 5 to 10 years [6] and each year, around 1,700 doctors leave general practice [7]. Conversely, 1,874 newly licensed GPs were certified in 2022, representing about 15% of all medical board certifications [6]. This has resulted in a decrease in the proportion of GPs over time, from 65.4% in 1979 [8] to 36% in 2022 [9]. It is projected that by 2025, there will be a shortage of approximately 20,000 GPs [10]. In order to achieve a 1:1 ratio of specialists to generalists in the outpatient sector, it would be necessary for approximately 25% of all board certifications to be in general practice.

Despite an increased interest in pursuing general practice among medical students in Germany, from 8.9% in 2014 to 11.2% in 2022 [11], challenges remain. Issues such as insufficient funding, bureaucratic hurdles, and regional differences in postgraduate training [11] contribute to these challenges, prompting various changes and innovations in the past decade. This article aims to describe the current structure and challenges of postgraduate training in general practice in Germany for both national and international readers.

## Methods

For this narrative review we completed a selective literature search in PubMed for articles published at any date regarding vocational training and postgraduate medical education/training for general practice as well as the role of general practitioners in Germany. Additionally, data from German institutions, such as the (National) Association of Statutory Health Insurance Physicians and medical associations, as well as legal texts, data from German medical journals not indexed in PubMed, and personal experiences of the authors in the field were included in our synthesis. Regarding terminology, we used official translations as available directly from the German institution concerned (e.g., state medical associations). Otherwise, we used translations provided by the National Association of Statutory Health Insurance Physicians or online dictionaries. Terms that were used in previous publications have been retained [5].

## Structure and organisation

### Role of GPs in the German healthcare system

Primary care in Germany is provided by three specialties: general practitioners, outpatient paediatricians, and outpatient general internal medicine specialists. General practice as a medical specialty was established in the former German Democratic Republic (GDR) in 1967 and in the Federal Republic of Germany (FRG) in 1972 [12], currently requiring at least five years of postgraduate training followed by a successful board certification [13], analogous to the training for most medical specialties. Historically, it was possible to register as a “practical doctor” (praktischer Arzt) [6], [9] six months after graduating from medical school and without completing postgraduate training. Although this pathway to primary care is no longer available, a small number of practical doctors remain in practice [4]. 

Patients in Germany can freely choose their GP without any limitation or mandatory registration [1], meaning they can have more than one GP simultaneously, often without the GPs being aware of each other. Although consultations with medical specialists should ideally be initiated by GP referrals, patients have direct access to specialists without needing a referral. Consequently, GPs do not serve a gate-keeping role [14] as they do in countries like Spain or the United Kingdom. Despite the higher proportion of specialists compared to generalists in ambulatory care [6], access to specialists is increasingly difficult due to high patient demand.

Outpatient paediatricians provide care exclusively to children. However, they can offer limited services to adults, such as additional vaccination of parents or continuous treatment for patients with specific medical conditions, such as those in paediatric cardiology [15]. In contrast, general practitioners, outpatient general internal medicine specialists, and practical doctors can and often do provide medical care for children. This is the case particularly in rural areas [16], where outpatient paediatric practices are not financially viable.

In addition to routine tasks during office hours, such as treating acute and chronic diseases, conducting check-ups and vaccinations, and handling bureaucratic tasks such as completing applications for rehabilitation or home care, many GPs also offer home or nursing home visits. Furthermore, GPs provide services after-hours during periodic on-call shifts on nights and weekends. These on-call shifts are mandatory for all Statutory Health Insurance(SHI)-authorised physicians working in the ambulatory sector (not just GPs) [17].

The regional distribution of GPs and specialists in ambulatory care is regulated by the state associations of statutory health insurance physicians (Kassenärztliche Vereinigung) according to federal guidelines, which is influenced by demographic, socioeconomic, and infrastructural factors. Many cities have the full number of GPs as stipulated by regulations while rural areas suffer from a shortage of GPs [18]. Billing for patients with a statutory health insurance is also handled by the state associations of statutory health insurance physicians [1]. Patients with private healthcare insurance typically front the costs of their care and are reimbursed according to their insurance policy [19].

### Prerequisites and application process to enter postgraduate training

After successfully completing medical school in Germany, junior doctors must apply for a medical license at a state examination office for medical professions (Landesprüfungsamt für Heilberufe) in the federal state where they completed medical school. Once licensed, they are free to choose a postgraduate training position in their desired medical specialty [5]. There is no matching process, pre-registration or central application system; instead, application and selection occur through a free-market approach. Junior doctors decide on the location and type of medical service in which they wish to work, whether in hospitals or ambulatory settings. Generally, there are no formal postgraduate training programs with planned rotation and a curriculum. Once they decide on a specialty, they sign up for the corresponding electronic logbook and officially register with the medical association in their state. A standardised, Germany-wide registration system of junior doctors does not exist. Due to the free-market (as opposed to program-based) postgraduate training system as well as the shortage of junior doctors across nearly every specialty, junior doctors can flexibly start, interrupt, or change workplaces and specialty at almost any time. Consequently, there is no workforce planning for junior doctors [5].

There are exceptions to the standard postgraduate training process for medical school graduates who were accepted by way of the “rural doctor quota” (Landarztquote) program. This initiative varies across states but generally offers medical school admission to applicants who may not meet the standard admission requirements but are committed to pursue postgraduate training in general practice and subsequently to working as a GP in a rural area for at least 5 to 10 years after board certification [20]. If a graduate of this program does not choose general practice for postgraduate training, penalties specified in their contract must be paid. These penalties vary among the federal states [21].

For junior doctors who did not graduate in Germany, especially those from countries outside the European Union, the application process for a medical license is more complicated on a bureaucratic level. Additional exams may be necessary to validate the qualifications of foreign medical graduates and to ensure their medical and language skills meet the standards required to practice medicine in Germany [5].

### General practice training

**Figure 1:**
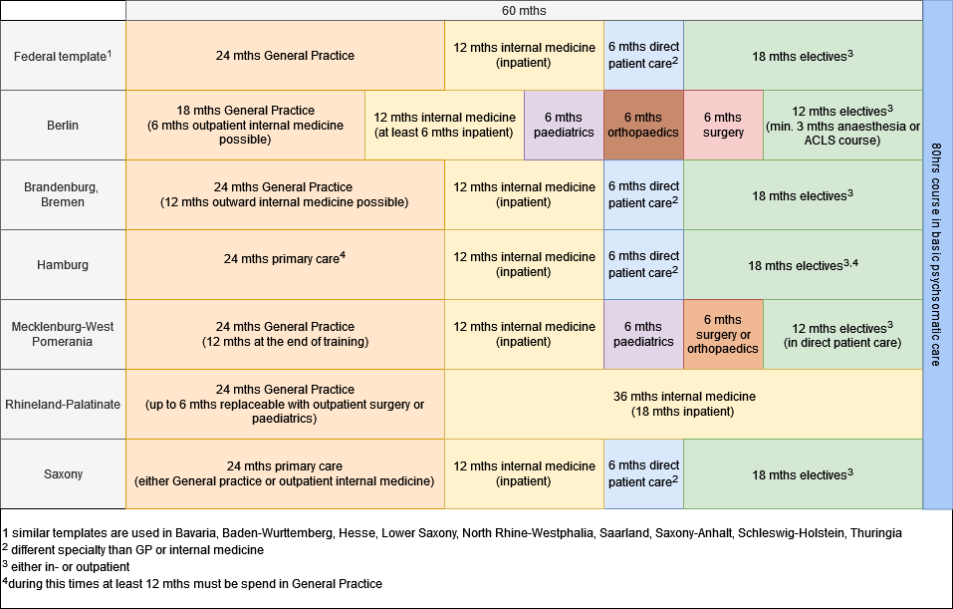
Required rotations for postgraduate training in general practice across the federal states of Germany. The sequence of these rotations is not specified (“mths”=months).

Postgraduate training in general practice in Germany has a minimum duration of 60 months and includes mandatory rotations in internal medicine and general practice. The specifics of these rotations are defined by a template for postgraduate training provided by the German Medical Association, last updated in 2018 [13]. However, due to the federal state system, each of the 17 German medical associations can modify this template to some extent, resulting in variations in the duration and content of required rotations across federal states. For example, some states have adopted the federal template, while others require specific periods in paediatrics, surgery, or anaesthesia. Figure 1 (Fig. 1) illustrates the requirements for each federal state. 

The order in which the required rotations for general practice are completed is not specified and is mostly influenced by job availability, personal interests, and individual circumstances of the junior doctors. Occasionally, local cooperations assist in coordinating these rotations, such as the coordination offices described below. Junior doctors must document every rotation and the acquired skills and knowledge in a logbook, which must be signed by their supervisors. The completed logbook later forms part of the basis for board certification and is examined and approved by the state medical association.

When choosing a job during postgraduate training, a junior doctor must ensure that the chosen facility or clinical supervisor is licenced to supervise their postgraduate training in their specialty of choice; otherwise, the rotations will not be recognised in the board certification process by the state medical associations. A license to supervise postgraduate training may be acquired from the state medical association by any doctor with a certain amount of work experience whose workspace meets specific structural and professional conditions, such as a sufficient patient flow and a representative number of typical diseases and disorders of the specialty. Didactic education or experience is generally not required, with the exception of Lower Saxony [22]. Hospital wards must provide certain diagnostic tests with a defined frequency to obtain a license. The workforce of junior doctors is essential for the majority of hospitals to ensure medical care on wards and in emergency rooms, especially during night and weekend shifts. Therefore, junior doctors are highly sought-after. However, hospitals are not required to provide additional training to junior doctors, such as courses, seminars, or simulations.

The logbook includes all specific topics outlined in the postgraduate training template of that state. Unlike other medical specialties, postgraduate training in general practice does not require a minimum number of procedures or examinations to be carried out. The logbook often serves more as a checklist rather than an actual assessment tool of competencies, in contrast to systems in other countries like the United Kingdom, where tools such as mini clinical evaluation exercises and direct observation of procedural skills are used for direct assessment by clinicians [5]. Beginning in 2020, there has been an online version of the logbook (eLogbook), but for rotations completed before this, the paper version is still accepted for board certification. Additionally, the logbook can be used to acknowledge competencies and rotations for a new specialty after a career change. 

The German College of General Practitioners and Family Physicians (DEGAM) has developed a competency-based curriculum for junior doctors and their supervisors to use on a voluntary basis to structure training in general practice [23], [24]. Unlike the eLogbook, this curriculum is divided into three main areas: medical knowledge (covering typical diseases and causes for consultations categorised by organ systems), personal competences (such as communication, management, and teaching) and procedures that reflect typical tasks of GPs. During rotations, the junior doctor and the supervisors re-evaluate the competencies acquired by the junior together over time. In practice, this competency-based curriculum is not universally adopted and its use heavily depends on the individual supervisor. 

A course in basic psychosomatic care is required in all federal states. The course totals 80 hours, consisting of 20 hours of theoretical education, 30 hours of communication training, and 30 hours of participation in Balint groups [25]. The aim is to equip the junior doctors with the skills needed to identify and manage psychosomatic diseases and comorbidities, improve the patient-doctor-relationship, and foster interdisciplinary collaboration [25]. The costs must be borne by junior doctors, although a refund may be possible after successful board certification in general practice [26] or via free offerings by competence centres [27].

In 2007, the first competence centre for general practice was founded as an association of the five medical schools in Baden-Wurttemberg. Its goal was to provide courses, seminars and/or simulations to establish a uniformly high standard in postgraduate training of GPs across the state. Between 2012 and 2016, North Rhine-Westphalia, Hesse, Thuringia, and Mecklenburg-West Pomerania also founded competence centres [28]. Today 16 different competence centres exist, two in North Rhine-Westphalia (North Rhine [29] and Westfalia-Lippe [30]) whereas Bremen has none [31], [32] but is part of the competence centre in Lower Saxony. Since 2017, funding of the competence centres by the statutory and private health insurances has been mandated by law [33], [34]. The main motivation of the legislators was to address the GP shortage by improving various aspects of postgraduate training. The competence centres offer workshops for junior doctors, mentoring programs, and train-the-trainer seminars supervisors of postgraduate medical training in general practice [35]. In 2022, approximately 2,700 junior doctors and 700 supervisors attended the seminars and workshops [36]. The regional implementation varies largely, reaching between 20% and 80% of junior doctors.

Given the complex rotation requirements for postgraduate training in general practice and free-market approach in Germany, organising the required rotations without gaps in between can be difficult and lead to interruptions and subsequent prolongation of training. To optimise the organisation of rotations, several federal states initiated coordination offices for GP training. Their aim is to coordinate regional funding for postgraduate training, offer information and job opportunities for junior doctors and training sites, and support so-called regional postgraduate training associations [33], [37]. In contrast, other federal states offer (online) job markets through organisations such as the state medical association, the state association of statutory health insurance physicians or the competence centres. Postgraduate training associations have been established in some federal states to improve the structure and organisation of postgraduate medical training in general practice. These typically include at least one hospital and various outpatient training sites, encompassing GPs as well as other medical specialties like surgery or paediatrics. The objective is to facilitate a continuous (gapless) training program that covers all required medical specialties, thereby minimising interruptions during training. This approach aims to eliminate the need for multiple job applications at the start of every rotation by using one contract for all sites within the association and to reduce the necessity for relocating or traveling long distances to training sites. Additionally, individual circumstances such as parental leave or working part-time are intended to be more easily accommodated with the help of a rotation coordinator employed by the training association [38]. 

In Baden-Wurttemberg, this concept was taken one step further. In 2009, the postgraduate training associations and the competence centre initiated the “Verbundweiterbildung^plus^” project, a structured network for postgraduate training, in which workforce planning within the members of the network enables pre-allocated job rotations. Additionally, junior doctors work and train within a coordinated curriculum. Junior doctors in this network are exempted from work to attend workshops and seminars at the competence centres, and funding is provided. Until 2017, there was a reported increase in the number of participants, both trainees and supervisors, and positive evaluations of the program were received [28].

### Subspecialty training options

In Germany, there are various types of subspecialties and additional qualifications [39], [40], [41] available. The most popular ones are naturopathy, emergency medicine, manual medicine and acupuncture [40]. The above mentioned course in basic psychosomatic care is a requirement for board certification in general practice, gynaecology and paediatrics. Others allow the provision and billing of additional services or treatments (e.g., acupuncture). Not all of them are acknowledged by the state medical associations as an official additional qualification (e.g., diving medicine) [39], [40], [41]. While some courses require completion of medical specialist training as a prerequisite (e.g., acupuncture, manual medicine) and can be obtained by attending weekend courses, others can or should be started during postgraduate training, as completing the requirements might be difficult otherwise. For instance, additional qualifications in allergology or diabetology can only be obtained by working for a period of time at a licensed training site. As a result, these qualifications cannot usually be acquired while running an outpatient practice.

On the other hand, while ultrasound examination skills may sometimes be assessed during board certification in general practice, completing a specific course is not mandatory for GP postgraduate training. However, to bill for ultrasound examinations, GPs must obtain additional qualifications, which involve specific courses and a required number of completed ultrasound examinations. Although these ultrasound courses are popular among various medical specialties (e.g., internal medicine, surgery), they are expensive and often not cost-effective for GPs due to high equipment costs and the time-intensive nature of ultrasound examinations [39]. 

### Career change

For junior doctors transitioning from another medical specialty to general practice during postgraduate training, the process is usually straightforward. Training periods involving direct patient care can typically be credited toward GP training, with any missing requirements fulfilled subsequently during GP training, in accordance with the standards set by the responsible medical association and prevailing funding conditions for state-funded training positions in ambulatory care [42].

Furthermore, a reduced training period may be granted if a doctor is already board-certified in a different specialty involving direct patient care (e.g., anaesthesia, surgery, gynaecology). However, the total of 24 months of training in general practice and the psychosomatic care course must be completed in order to be eligible for board certification [43], [44], [45]. The amount of credited training time varies among the different state medical associations. Shortly after its implementation in 2011, this model of a shortened training pathway was found to be most popular among former anaesthesiologists (37.5%) and surgeons (25%) seeking to become GPs [46].

The pathway for internal medicine specialists to primary care represents an exception to the scenario above. These specialists can apply to work directly as primary care providers in outpatient clinics without prior training in ambulatory care. Therefore, state funding of additional outpatient training (see below) is not always granted in these cases [42].

### Income during GP training

Junior doctors are either paid by the hospital or the (self-employed ambulatory) doctor who acts as employer and training supervisor. While hospitals are financially able to pay junior doctors from hospital revenues, outpatient practices cannot simply increase their income temporarily to accommodate an additional salary, e.g. by seeing more patients. Therefore, the salary for junior doctors in the ambulatory sector is subsidised. In 2016, the German Hospital Association, the National Association of Statutory Health Insurance and the National Association of Statutory Health Insurance Funds, in agreement with the Association of Private Health Insurance and the German Medical Association, agreed to fund a portion of postgraduate medical training in ambulatory care [33]. By law, at least 7,500 full-time positions in general practice must be funded annually by the state, encompassing both outpatient and inpatient care. The Association of Statutory Health Insurance cannot impose a limit on funding for ambulatory care. Furthermore, each rotation must last a minimum of three months to qualify for state funding [33] and the funding is granted for the minimum duration of postgraduate training. If parts of the training were completed in the training for another medical specialty, no additional funding is available. For example, if a junior doctor worked in inpatient internal medicine for two years before transitioning to general practice afterwards, the junior doctor will not receive any more funding for training positions in internal medicine. However, any missing rotations for GP training (such as paediatrics or general practice) will be funded [33].

Due to organisational hurdles, gaps of time between the completion of GP training and board certification are common. In exceptional cases, state funding for this interim period may be possible [33]. However, if a doctor fails the board certification exam and requires additional training to qualify for a second attempt, no funding is granted [33]. This can cause significant financial and organisational difficulties.

#### Income during inpatient rotations

Inpatient jobs for GP trainees are primarily funded by statutory and private health insurances. Any difference between this and the salary specified by collective agreements is paid directly by the hospital [33]. Collective agreements typically stipulate that salaries for junior doctors increase with each year of postgraduate training, reflecting cumulative years of experience. This incremental salary structure applies to doctors in GP training as well, even if their previous training was in an outpatient setting or at a different hospital.

#### Income and funding during outpatient rotations

GP trainees in outpatient settings are also typically paid via funding from statutory and private health insurances, supplemented by additional funding from the Association of Statutory Health Insurance Physicians. In medically underserved areas or those at risk of such, bonuses are provided to further support these positions [33]. The aim of this funding structure is to align outpatient salaries with those of inpatient jobs. Unlike inpatient positions, outpatient salaries do not commonly increase based on years of experience. However, the actual salary can be individually negotiated with the outpatient employer and supervisor, potentially leading to better income.

### Board certification

After completing all requirements, GP trainees can apply for board certification. The state medical association will review the completion of rotations and the logbook [5]. If all requirements are met, the date for the individual oral exam will be provided at least 14 days in advance. The examination committee for GP certification consists of at least two GPs and one other medical specialist [13]. The exam takes between 30 [16], [47], [48] and 60 minutes [49]. Possible content includes patient cases, interpretation of electrocardiograms, pictures of typical medical findings or ultrasound examinations [50]. Examiners are not required to have completed specific preparatory courses or didactic qualifications. There are no officially defined exam topics or catalogues, and basic requirements for passing the exam can vary between different state medical associations [50].

### Following board certification

Successful board certification is a prerequisite for applying for registration with the Association of Statutory Health Insurance Physicians [51]. Without this registration, billing for patients with statutory health insurances, which covers 90% of the population, is not possible [52]. 

In Germany, the majority of GPs are self-employed, either in private or group practices. However, a growing number are now working as employed doctors in medical care centres, hospitals, or for insurance companies [6]. For those aspiring to self-employment in certain urban areas already saturated with GPs, securing a statutory health insurance practice can be challenging. To facilitate transitions, some owners of private practices employ young colleagues who will eventually take over the practice upon their retirement [18].

After board certification, joining a group practice was the most popular option for GPs. However, female doctors were found to prefer employment over managing their own private practice, while male doctors favoured the latter [53]. Recent studies have also highlighted an increasing proportion of female junior doctors [7], [53]. Furthermore, 44.2% of female and 41.9% male doctors have children, with 25.3% of women working part-time compared to 2.2% of men. Regardless of sex, there is a growing emphasis on part-time work and improving work-life-balance. This trend has contributed to a shortage of available working hours. Between 2011 and 2021, the average working time decreased from 50 to 48.5 hours per week, and the number of employed doctors increased from approximately 19,600 in 2012 to around 46,000 in 2022 [54], while the number of self-employed doctors decreased. Evaluations among current medical students suggest that these trends will continue [11].

While there is no recertification process like in countries such as the United Kingdom, continuing medical education (CME) is required for all doctors to maintain medical competence and standards. Every five years, the fulfilment of CME requirements is checked and certified by the state medical boards [55].

## Advantages and disadvantages of general practice as an aspired specialty

When asked to provide reasons to pursue or not pursue general practice for postgraduate training, medical students and junior doctors often cited similar factors [11], [53]. One significant reason is the structure of the training itself. For postgraduate training in inpatient care specialties, trainees typically remain at a single hospital, ensuring continuity throughout their training period with contracts that cover its entirety. This fosters an environment in which senior doctors are more likely to supervise and support junior doctors over an extended period of time, sometimes extending beyond board certification. This also facilitates seamless extensions of rotations in case of illness or parental leave. Collective agreements further ensure that salaries increase progressively, even beyond the mandated training duration.

On the contrary, general practice requires trainees to navigate multiple job changes, necessitating frequent job applications and introducing uncertainties regarding securing suitable employment at the right time and location. This can result in long commutes or periods of unemployment if positions are not readily available [38], [56]. Registering for unemployment, meanwhile, entails navigating bureaucratic procedures, is time-consuming, and may carry a social stigma. However, without ongoing employment, junior doctors must cover living expenses as well as personal contributions for health and pension insurance. 

On the other hand, the training template for general practice offers a degree of flexibility that allows for personalised career paths. Apart from mandatory periods in inpatient care, GP trainees can predominantly focus on rotations within the ambulatory sector and select elective areas aligned with their personal interests. The attractiveness of outpatient care among medical students and young junior doctors is due to its flat hierarchies and better compatibility with family responsibilities [11], [53]. Unlike hospital settings, where trainees are often required to cover nights, weekends, and public holidays, outpatient practices typically operate within regular office hours. After-hours services are managed by designated practices or on-call doctors. Junior doctors can participate in these services on a voluntary basis, which results in opportunities to gain experience and earn additional income without mandatory involvement in shifts.

However, securing rotations in certain medical specialties can be challenging for junior doctors, particularly in rural areas with limited licensed supervisors, for example in paediatrics or Dermatology. In situations where multiple junior doctors are vying for a single training position at a paediatric practice, careful planning and sometimes dividing rotations into smaller durations may be necessary. Moreover, moving between federal states can also pose challenges regarding the acceptance of previously completed rotations by the new state medical association and continuity in funding [56]. The KarMed-Study highlighted that general practice had the lowest proportion of board-licensed doctors after 10 years of postgraduate training, indicating that GP trainees require longer periods of time to complete their training [57].

Following board certification, concerns often revolve around financial planning and penalties for exceeding budgets, substantial bureaucratic responsibilities, high workload, and insufficient funding compared to other medical specialties. Moreover, the restrictions as regulated by the state associations of statutory health insurance physicians on the number of practices that can be established in desirable locations [11], [53] dissuade many from pursuing a career as an outpatient-based doctor, irrespective of their desired specialty [11].

## Conclusion

Postgraduate training for general practice in Germany is complex and differs significantly between the federal states and when compared to other countries. Recent years have seen numerous improvements in funding, training, and working conditions for general practice trainees. While on-the-job training is important, it must be complemented by adequate supervision and support by way of seminars, lectures, and workshops provided by competence centres. These centres also offer regular didactic education for supervisors of postgraduate medical training, which is currently mandatory only in Lower Saxony; hopefully, other federal states will adopt this practice. The 2018 update of the federal training template, including the eLogbook, and the competence-based curriculum developed by DEGAM mark initial steps away from purely numerical requirements towards the actual acquisition of relevant skills during postgraduate medical training in general practice. Improved funding and support for organising rotations could mitigate the disadvantages associated with general practice when compared to other specialties with exclusively inpatient training.

Nevertheless, challenges remain. The improvements that have been made to date have not attracted enough new GP trainees to compensate for the annual retirement of general practitioners [58]. Increasing the exposure of medical students to primary care could increase their interest in this field. There is also a need for the expansion of the scope of the competence centres and the development of more structured training programmes. The competence centres should not only focus on educational activities, but also manage administrative and workforce planning responsibilities. Mandating participation in curricular training, closer supervision of junior doctors, and assistance in the organisation of rotations at various sites could shorten training and improve board certification success rates [58]. This would help address the GP shortage by enabling trainees to assume responsibility for their own practice sooner [59].

In conclusion, while enhancing GP training and increasing the attractiveness of general practice are crucial for recruiting new professionals, these measures alone may not suffice. Comprehensive workforce planning is essential, albeit challenging, for securing adequate primary care provision in the future. Unfortunately, this proposal has already been rejected by the German Medical Association.

## Notes

### Notes on the contributors

JFC is GP and Head of the Department of General Practice at the University Medicine Greifswald.

JFMP completed part of postgraduate medical training in the UK and Germany and is a doctoral researcher in Denmark.

EM and CT completed part of postgraduate training and are currently junior doctors in Mecklenburg-Vorpommern, Germany.

### Authors’ ORCIDs

- Jean-François Chenot: 0000-0001-8877-2950
- Julia Freyer Martins Pereira: 0000-0001-6119-0651
- Elizabeth Mathias: 0000-0003-3679-671X
- Caroline Tornow: 0009-0002-8754-8345

### Competing interests

The authors declare that they have no competing interests.
